# Supplementary material for: Factors associated with under-reporting of head and neck squamous cell carcinoma in cause-of-death records: A comparative study of two national databases in France from 2008 to 2012
Source: PLoS One. 2021 Feb 3;16(2):e0246252. doi: 10.1371/journal.pone.0246252 (PMC7857613; doi:10.1371/journal.pone.0246252)
Supplement: S1 File — (DOCX) [file pone.0246252.s001.docx]

**Factors associated with under-reporting of head and neck squamous cell carcinoma in cause-of-death records: a comparative study of two national databases in France from 2008 to 2012.**

**Supplementary material contents**

[S1 Methods: Imputation of mortality outside hospital 2](#_Toc56512232)

[Overview 2](#_Toc56512233)

[Methods 2](#_Toc56512234)

[Morbidity profile of patients with HNSCC discharged in 2008 3](#_Toc56512235)

[Imputation of mortality outside hospital from 2008 to 2013 3](#_Toc56512236)

[Table 1. Morbidity profile of patients discharged with HNSCC in 2008, by vital status (N=40,811) 5](#_Toc56512237)

[Table 2. Risk of in-hospital death in 2008 among patients with HNSCC and known vital status in 2008 (N=35 515) 8](#_Toc56512238)

[Table 3. Vital status of patients discharged with HNSCC, by year 10](#_Toc56512239)

[S1 Table: Coding dictionary 11](#_Toc56512240)

[S2 Table: EPICORL study population 14](#_Toc56512241)

[S3 Table: Identification of deaths attributable to HNSCC in the PMSI database, overall and by year of death (France, 2008-2012) 15](#_Toc56512242)

[S4 Table: Characteristics of deaths attributable to HNSCC only identified in the PMSI database (France, 2008–2012) 16](#_Toc56512243)

## S1 Methods: Imputation of mortality outside hospital

### Overview

Hospital admission remains frequent at end-of-life in France, overall and for cancer patients in particular. A majority of deaths occur at hospital, with stable figures over the last three decades [[1](#_ENREF_1_1)]. In addition, about two-third patients receive hospital care in the month preceding death [[2](#_ENREF_1_2),[3](#_ENREF_1_3)]. In cancer patients, the role of hospital is even exacerbated at end-of-life: death was recorded at hospital for 73% deceased patients in 1993-2008, without changes over time [[4](#_ENREF_1_4)], and above 90% patients received hospital care in the month preceding death in 2009 [[2](#_ENREF_1_2),[3](#_ENREF_1_3)]. We relied on this particular feature of the French healthcare system to impute mortality outside hospital among patients lost to follow-up based on their morbidity profile at last hospital discharge [[5](#_ENREF_1_5)].

### Methods

The French National Hospital Discharge (PMSI) database contains all public and private hospital claims for acute and post-acute care from 2008 to 2013. Accordingly, the vital status of each patient diagnosed with HNSCC can be followed with use all his/her admissions to hospital until in-hospital death or last hospital discharge in 2008-2013. In patients alive at last hospital discharge, all hospital records are informative on their morbidity profile and therefore immediate mortality risk outside hospital [[6](#_ENREF_1_6)].

We used a sequential approach by year of hospital admission to impute mortality outside hospital among all patients with a diagnosis of HNSCC and lost to follow-up in the same year. Expectedly, patients admitted to hospital in 2008 had the lowest level of attrition as they may be followed at hospital over the next five years (2009-2013). Therefore, we characterized the morbidity profile of patients with known vital status in 2008 with use of all hospital records of 2008 and estimated the probability of death in a given year with use of multivariate logistic regression. Then we imputed mortality outside hospital at an optimal cut-off (min $\left\{ \sqrt{{(1-specificity)}^{2}+{(1-sensitivity)}^{2}} \right\}$) based on the morbidity profile of patients lost to follow-up at hospital from 2008 to 2013. Finally, because the proportion of patients lost to follow-up at hospital increases exponentially in 2013, we censored all information on vital status at July 1, 2013 in the study population selected in 2010-12, i.e., after the initial treatment period of six months necessary to characterize patients first diagnosed with HNSCC in December 2012.

To characterize morbidity profiles, a large amount of information was retrieved from all hospital records in a given year:

1. *Demographics*: gender, age in January, zip code of residency categorized in French regions.
2. *Primary cancer other than HNSCC*: lung cancer, esophageal cancer, and any other primary cancer [[7](#_ENREF_1_7)].
3. *Cancer stage*: distant metastasis (ICD-10 C78-C79), and otherwise lymph nodes (ICD-10 C77).
4. *Causes-of-death other than cancer.* We relied on the Global Burden of Disease study methodology to disentangle ICD-10 codes acceptable as an underlying cause-of-death from “garbage codes” [[8](#_ENREF_1_8),[9](#_ENREF_1_9)]. Acceptable causes-of-death and conditions that are intermediate causes of death (e.g., ICD-10 N17 for acute renal failure) were recorded by ICD-10 chapter. In absence of an acceptable cause-of-death, conditions that cannot be considered as the underlying cause-of-death were recorded by ICD-10 chapter (e.g., ICD-10 K02 codes for dental caries).
5. *Other prognostic factors*. Several factors of ICD-10 chapters XVIII and XXI were recorded: palliative care (ICD-10 Z51.5); chronic dialysis (medical procedures); transplantation status (ICD-10 Z94; T86 and medical procedures); poor general condition (ICD-10 R53.+0); shock (ICD-10 R57); bedridden (ICD-10 R26..30 or Z74.00); senility (ICD-10 R54).
6. *Hospital trajectory in the year*: total length of stay in acute inpatient care (in nights); admission in acute day case care (without inpatient care admission); emergency room admission; post-acute care admission; day case care at home; and nursing home admission.

In preliminary analyses, we found that patients lost to follow-up in palliative care or chronic dialysis had dramatic immediate mortality risks. Of 30 238 patients recorded in palliative care before October 2013, in-hospital death rate was 90% within 3 months. Of 618 patients on chronic dialysis at last discharge before October 2013, in-hospital death rate was 73%. We assumed that these patients lost to follow-up in palliative care or chronic dialysis before October 2013 all died immediately after hospital discharge.

### Morbidity profile of patients with HNSCC discharged in 2008

Of 40,811 patients discharged with HNSCC in 2008, 8750 (21.4%) died at hospital in 2008, 26,765 (65.6%) were alive by the end of 2008 (i.e., they had hospital records in 2009-2013), and 5296 (13.0%) were lost to follow-up at hospital in 2008 (**Table 1**). Patients lost to follow-up in 2008 were rather in better health than patients alive by the end of 2008 and even more so when compared to patients who died in 2008.

### Imputation of mortality outside hospital from 2008 to 2013

**Table 2** shows the results of the multivariate logistic regression on the probability of in-hospital death among 35,515 patients with known vital status in 2008. Overall, the concordance reached 88.5% between observed and predicted deaths in 2008. At the optimal cut-off (i.e., 20.5% predicted probability of death), sensitivity and specificity were 79.8% and 76.9%, respectively.

Mortality outside hospital was imputed at the optimal cut-off based on the morbidity profile of patients lost to follow-up at hospital from 2008 to 2013 (**Table 3**). Of 5296 patients lost to follow-up at hospital in 2008, 1450 (27.4%) deaths were imputed. While the proportion of patients lost to follow-up at hospital increased slightly from 2008 to 2012, it increased exponentially in 2013 and vital status was censored at December 31, 2012.

Reference List

1. Institut national de la statistique et des études économiques (2014) Répartition des décès selon le lieu du décès [Distribution of deaths according to location]. Institut national de la statistique et des études économiques.

2. Pennec S, Monnier A, Pontone S, Aubry R (2012) End-of-life medical decisions in France: a death certificate follow-up survey 5 years after the 2005 act of parliament on patients' rights and end of life. BMC Palliat Care 11: 25.

3. Observatoire National de la Fin de Vie (2013) Rapport 2012: vivre la fin de vie chez soi. [2012 Report: living end-of-life at home]. <http://www.ladocumentationfrancaise.fr/rapports-publics/134000186-observatoire-national-de-la-fin-de-vie-rapport-2012-vivre-la-fin-de-sa-vie-chez-soi>. Observatoire National de la Fin de Vie,. 143 p.

4. Gisquet E, Aouba A, Aubry R, Jougla E, Rey G (2012) Où meurt-on en France ? Analyse des certificats de décès (1993-2008) [Where do we die in France? Analysis of death certificates (1993-2008)]. Bulletin Epidémiologique Hebdomadaire 48: 547-551.

5. Schwarzinger M, Baillot S, Yazdanpanah Y, Rehm J, Mallet V (2017) Contribution of alcohol use disorders on the burden of chronic hepatitis C in France, 2008-2013: A nationwide retrospective cohort study. J Hepatol 67: 454-461.

6. Schildcrout JS, Basford MA, Pulley JM, Masys DR, Roden DM, et al. (2010) An analytical approach to characterize morbidity profile dissimilarity between distinct cohorts using electronic medical records. J Biomed Inform 43: 914-923.

7. Jegu J, Colonna M, Daubisse-Marliac L, Tretarre B, Ganry O, et al. (2014) The effect of patient characteristics on second primary cancer risk in France. BMC Cancer 14: 94.

8. Lozano R, Naghavi M, Foreman K, Lim S, Shibuya K, et al. (2012) Global and regional mortality from 235 causes of death for 20 age groups in 1990 and 2010: a systematic analysis for the Global Burden of Disease Study 2010. Lancet 380: 2095-2128.

9. Naghavi M, Makela S, Foreman K, O'Brien J, Pourmalek F, et al. (2010) Algorithms for enhancing public health utility of national causes-of-death data. Popul Health Metr 8: 9.

###

### Table 1. Morbidity profile of patients discharged with HNSCC in 2008, by vital status (N=40,811)

| **Morbidity profile** | | **In-hospital death in 2008** | **Alive by the end of 2008** | **Lost to follow-up in 2008** | **P-value** |
| --- | --- | --- | --- | --- | --- |
|  |  | **N=8,750** | **N=26,765** | **N=5,296** |  |
| **Men** | | 7,289 (83.3) | 21,725 (81.2) | 4,195 (79.2) | <0.0001 |
| **Age in January 2008, mean (std) years** | | 64.3 (11.9) | 61.4 (11.5) | 61.5 (12.8) | <0.0001 |
| **French Main region** | |  |  |  |  |
|  | Greater Paris region | 1,337 (15.3) | 3,885 (14.5) | 818 (15.5) | <0.0001 |
|  | North-West | 2,268 (25.9) | 6,454 (24.1) | 1,516 (28.6) |  |
|  | North-East | 2,376 (27.2) | 7,117 (26.6) | 1,203 (22.7) |  |
|  | South-West | 819 (9.4) | 2,587 (9.7) | 467 (8.8) |  |
|  | South-East | 1,893 (21.6) | 6,555 (24.5) | 1,247 (23.6) |  |
|  | French West Indies | 57 (0.7) | 167 (0.6) | 57 (0.9) |  |
| **Other cancer (by ICD-10 chapter)** | |  |  |  |  |
|  | Lung cancer (II) | 1,072 (12.3) | 1,474 (5.5) | 207 (3.9) | <0.0001 |
|  | Esophageal cancer (II) | 531 (6.1) | 809 (3.0) | 115 (2.2) | <0.0001 |
|  | Cancer other than HNSCC, lung, or esophageal (II) | 1,688 (19.3) | 3,875 (14.5) | 606 (11.4) | <0.0001 |
|  | Metastasis (II) | 3,316 (37.9) | 2,345 (8.8) | 518 (9.8) | <0.0001 |
|  | Lymph nodes (without metastasis) (II) | 1,143 (13.1) | 4,314 (16.1) | 706 (13.3) | <0.0001 |
| **Other causes-of-death (by ICD-10 chapter)** | |  |  |  |  |
|  | HIV/AIDS (I) | 40 (0.5) | 115 (0.4) | 12 (0.2) | 0.078 |
|  | Infectious diseases other than HIV (I) | 2,506 (28.6) | 3,167 (11.8) | 573 (10.8) | <0.0001 |
|  | Blood disorders (III) | 1,964 (22.5) | 2,725 (10.2) | 482 (9.1) | <0.0001 |
|  | Malnutrition (IV) | 2,840 (32.5) | 3,915 (14.6) | 714 (13.5) | <0.0001 |
|  | Endocrine and metabolic disorders (IV) | 2,291 (26.2) | 6,073 (22.7) | 872 (16.5) | <0.0001 |
|  | Mental and behavioral disorders (V) | 2,578 (29.5) | 8,485 (31.7) | 1,326 (25.0) | <0.0001 |
|  | Diseases of the nervous system (VI) | 968 (11.1) | 1,589 (5.9) | 298 (5.6) | <0.0001 |
|  | Diseases of the circulatory system (IX) | 2,525 (28.9) | 6,183 (23.1) | 912 (17.2) | <0.0001 |
|  | Diseases of the respiratory system (X) | 2,086 (23.8) | 6,084 (22.7) | 936 (17.7) | <0.0001 |
|  | Diseases of the liver (XI) | 634 (7.3) | 1,257 (4.7) | 184 (3.5) | <0.0001 |
|  | Other diseases of the digestive system (XI) | 1,978 (22.6) | 5,316 (19.9) | 744 (14.1) | <0.0001 |
|  | Diseases of the skin and subcutaneous tissue (XII) | 551 (6.3) | 703 (2.6) | 125 (2.4) | <0.0001 |
|  | Diseases of the musculoskeletal system and connective tissue (XIII) | 333 (3.8) | 887 (3.3) | 111 (2.1) | <0.0001 |
|  | Diseases of the urinary system (XIV) | 473 (5.4) | 1,026 (3.8) | 129 (2.4) | <0.0001 |
|  | Gynecological diseases (XIV) | 6 (0.1) | 52 (0.2) | 10 (0.2) | <0.05 |
|  | External causes of mortality (XX) | 912 (10.4) | 1,963 (7.3) | 286 (5.4) | <0.0001 |
| **Intermediate causes-of-death (by ICD-10 chapter)** | |  |  |  |  |
|  | Infectious diseases other than HIV (I) | 607 (6.9) | 484 (1.8) | 91 (1.7) | <0.0001 |
|  | Endocrine and metabolic disorders (IV) | 841 (9.6) | 813 (3.0) | 159 (3.0) | <0.0001 |
|  | Diseases of the circulatory system (IX) | 555 (6.3) | 662 (2.5) | 92 (1.7) | <0.0001 |
|  | Diseases of the respiratory system (X) | 1,556 (17.8) | 1,140 (4.3) | 230 (4.3) | <0.0001 |
|  | Diseases of the liver (XI) | 183 (2.1) | 129 (0.5) | 19 (0.4) | <0.0001 |
|  | Diseases of the urinary system (XIV) | 804 (9.2) | 973 (3.6) | 165 (3.1) | <0.0001 |
| **Other prognostic factors (ICD-10 chapters XVIII and XXI)** | |  |  |  |  |
|  | Transplantation status | 31 (0.4) | 140 (0.5) | 11 (0.2) | <0.01 |
|  | Poor health condition | 2,568 (29.4) | 1,818 (6.8) | 428 (8.1) | <0.0001 |
|  | Shock | 2,022 (23.1) | 1,215 (4.5) | 208 (3.9) | <0.0001 |
|  | Bedridden | 515 (5.9) | 133 (0.5) | 52 (1.0) | <0.0001 |
|  | Senility | 48 (0.6) | 36 (0.1) | 7 (0.1) | <0.0001 |
| **Hospital trajectory (admission)** | |  |  |  |  |
|  | Inpatient care | 8,423 (96.3) | 23,694 (88.5) | 4,356 (82.3) | <0.0001 |
|  | Day case admission without inpatient care | 3,443 (39.4) | 13,013 (48.6) | 1,991 (37.6) | <0.0001 |
|  | Emergency room | 3,765 (43.0) | 5,027 (18.8) | 869 (16.4) | <0.0001 |
|  | Post-acute care | 2,033 (23.2) | 3,845 (14.4) | 553 (10.4) | <0.0001 |
|  | Day case care at home | 974 (11.1) | 784 (2.8) | 74 (1.4) | <0.0001 |
|  | Nursing home | 302 (3.5) | 361 (1.4) | 181 (3.4) | <0.0001 |

### Table 2. Risk of in-hospital death in 2008 among patients with HNSCC and known vital status in 2008 (N=35 515)

| **Morbidity profile** | | **Adjusted odds-ratio (95% CI)** | **P-value** |
| --- | --- | --- | --- |
| Men | | 1.15 (1.06-1.25) | <0.001 |
| Age | | 1.25 (1.13-1.38) | <0.0001 |
| Age^2^ | | 1.00 (0.99-1.00) | <0.0001 |
| Age^3^ | | 1.00 (1.00-1.00) | <0.0001 |
| French region (reference: Greater Paris region) | |  |  |
|  | North-West | 1.28 (1.16-1.42) | <0.0001 |
|  | North-East | 1.12 (1.02-1.24) |  |
|  | South-West | 1.18 (1.04-1.33) |  |
|  | South-East | 1.11 (1.00-1.22) |  |
|  | French West Indies | 1,43 (1,01-2,03) |  |
| **Other cancer (by ICD-10 chapter)** | |  |  |
|  | Lung cancer (II) | 1.22 (1.10-1.36) | <0.001 |
|  | Esophageal cancer (II) | 1.42 (1.24-1.64) | <0.0001 |
|  | Cancer other than HNSCC, lung or esophageal (II) | 0.93 (0.86-1.01) | 0.073 |
|  | Metastasis (II) | 5.37 (4.98-5.79) | <0.0001 |
|  | Lymph nodes (without metastasis) (II) | 1.32 (1.21-1.43) | <0.0001 |
| **Other causes-of-death (by ICD-10 chapter)** | |  |  |
|  | HIV/AIDS (I) | 1.03 (0.65-1.63) | 0.90 |
|  | Infectious diseases other than HIV (I) | 1.46 (1.35-1.58) | <0.0001 |
|  | Blood disorders (III) | 1.31 (1.20-1.42) | <0.0001 |
|  | Malnutrition (IV) | 1.55 (1.44-1.66) | <0.0001 |
|  | Endocrine and metabolic disorders (IV) | 0.76 (0.71-0.82) | <0.0001 |
|  | Mental and behavioral disorders (V) | 0.72 (0.67-0.78) | <0.0001 |
|  | Diseases of the nervous system (VI) | 1.18 (1.06-1.32) | <0.01 |
|  | Diseases of the circulatory system (IX) | 0.90 (0.84-0.97) | <0.01 |
|  | Diseases of the respiratory system (X) | 0.97 (0.90-1.05) | 0.48 |
|  | Diseases of the liver (XI) | 1.64 (1.44-1.85) | <0.0001 |
|  | Other diseases of the digestive system (XI) | 0.72 (0.67-0.78) | <0.0001 |
|  | Diseases of the skin and subcutaneous tissue (XII) | 1.42 (1.23-1.65) | <0.0001 |
|  | Diseases of the musculoskeletal system and connective tissue (XIII) | 0.69 (0.58-0.81) | <0.0001 |
|  | Diseases of the urinary system (XIV) | 0.62 (0.54-0.72) | <0.0001 |
|  | Gynecological diseases (XIV) | 0.30 (0.11-0.81) | <0.05 |
|  | External causes of mortality (XX) | 0.77 (0.69-0.86) | <0.0001 |
| **Intermediate causes-of-death (by ICD-10 chapter)** | |  |  |
|  | Infectious diseases other than HIV (I) | 1.93 (1.65-2.25) | <0.0001 |
|  | Endocrine and metabolic disorders (IV) | 0.72 (0.63-0.83) | <0.0001 |
|  | Diseases of the circulatory system (IX) | 1.55 (1.34-1.80) | <0.0001 |
|  | Diseases of the respiratory system (X) | 2.83 (2.56-3.14) | <0.0001 |
|  | Diseases of the liver (XI) | 2.57 (1.95-3.40) | <0.0001 |
|  | Diseases of the urinary system (XIV) | 1.19 (1.05-1.35) | <0.01 |
| **Other prognostic factors (ICD-10 chapters XVIII and XXI)** | |  |  |
|  | Transplantation status | 0.76 (0.47-1.24) | 0.27 |
|  | Poor health condition | 3.20 (2.95-3.46) | <0.0001 |
|  | Shock | 3.96 (3.57-4.40) | <0.0001 |
|  | Bedridden | 4.77 (3.82-5.97) | <0.0001 |
|  | Senility | 1.87 (1.14-3.08) | <0.05 |
| **Hospital trajectory (admission)** | |  |  |
|  | Inpatient care | 1.39 (1.22-1.59) | <0.0001 |
|  | Day case admission without inpatient care | 0.54 (0.51-0.58) | <0.0001 |
|  | Emergency room | 2.15 (2.01-2.29) | <0.0001 |
|  | Post-acute care | 1.11 (1.03-1.20) | <0.05 |
|  | Daycare at home | 3.06 (2.71-3.45) | <0.0001 |
|  | Nursing home | 1.42 (1.17-1.72) | <0.001 |

### Table 3. Vital status of patients discharged with HNSCC, by year

| **Year** | **Total patients discharged in the year** | **New patients in the year** | **In-hospital deaths in the year** | **Patients alive at the end of the year** | **Patients lost to follow-up in the year** | | | **Increase in overall deaths after imputation** |
| --- | --- | --- | --- | --- | --- | --- | --- | --- |
|  |  |  |  |  | **Total** | **Imputation of death status** | **Imputation of alive status** |  |
| 2008 | 40,811 | 40,811 (100) | 8,750 (21.4) | 26,765 (65.6) | 5,296 (13.0) | 1,450 (27.4) | 3,846 (72.6) | 1.17 |
| 2009 | 47,008 | 26,232 (55.8) | 9,693 (20.6) | 30,747 (65.4) | 6,568 (14.0) | 1,692 (25.8) | 4,876 (74.2) | 1.17 |
| 2010 | 50,224 | 23,158 (46.1) | 10,142 (20.2) | 32,345 (64.4) | 7,737 (15.4) | 1,793 (23.2) | 5,944 (76.8) | 1.18 |
| 2011 | 52,964 | 21,647 (40.9) | 10,372 (19.5) | 21,195 (60.8) | 10,397 (19.6) | 2,146 (20.6) | 8,251 (79.4) | 1.21 |
| 2012 | 55,074 | 20,505 (37.2) | 10,396 (18.8) | 28,521 (51.8) | 16,157 (29.3) | 2,922 (18.1) | 13,235 (81.1) | 1.28 |
| 2013 | 56,938 | 19,598 (34.4) | 10,383 (18.2) | 404 (0.7) | 46,151 (81.1) | 10,732 (23.3) | 35,419 (76.7) | 2.03 |

## S1 Table: Coding dictionary

| **Variables** | | | **International Classification of Diseases and Related Health Problems, 10^th^ Revision, French version (ICD-10-FR)** | **References** |
| --- | --- | --- | --- | --- |
| **Primary HNSCC site or UCoD** | | | | |
| Primary discharge diagnosis recorded at first HNSCC surgery or panendoscopy | | | | [[1](#_ENREF_5_1),[2](#_ENREF_5_2)] |
|  | Nasopharynx | | C11 |  |
|  | Nasal cavity or paranasal sinuses | | C30.0; C31 |  |
|  | Lip | | C00 |  |
|  | Tongue | | C01-C02 |  |
|  | Oral cavity | | C03-C06 |  |
|  | Oropharynx | | C09-C10 |  |
|  | Hypopharynx | | C12-C13 |  |
|  | Larynx | | C32 |  |
|  | Ill-defined HNSCC | | C14 |  |
| **Criteria used to define HNSCC as a probable/possible cause of death at hospital** | | | | |
| Stage at initial treatment (assessed within 6 months after first diagnosis of HNSCC) | | | | [[2](#_ENREF_5_2)] |
|  | Metastatic stage (any record of distant metastasis) | | C78-C79 |  |
|  | Locally advanced stage | |  |  |
|  |  | Any ICD-10 record indicating locoregional extension | C00.8; C02.8; C04.8; C05.8; C06.8; C08.8; C09.8; C10.8; C11.8; C13.8; C14.0; C14.8; C77 |  |
|  |  | Any treatment record eliminating an early stage | chemotherapy (Z51.1) surgery (HNSCC surgical procedures) and radiotherapy (Z51.0) tracheostomy at index HNSCC diagnosis (Z93.0, Z43.0, J95.0) palliative care (Z51.5) | [[3](#_ENREF_5_3),[4](#_ENREF_5_4)] |
|  | Early stage, by default | | -- |  |
| Relapse assessed after 6 months in patients treated at early or locally advanced stage | | | | [[2](#_ENREF_5_2)] |
|  | Local relapse | | Same primary discharge diagnosis |  |
|  | Any new event indicating extension | | distant metastasis locoregional extension chemotherapy |  |
| **Other cancer characteristics** | | | | |
| Multiple primary HNSCC | | |  | [[5-7](#_ENREF_5_5)] |
|  | Personal history of HNSCC at first diagnosis | | Z85.802; Z85.20 |  |
|  | Second synchronous HNSCC | | Any record at a different primary HNSCC site within 6 months after first diagnosis of HNSCC |  |
|  | Second metachronous HNSCC | | Any record at a different primary HNSCC site after 6 months |  |
| Primary cancer other than HNSCC | | |  | [[5-7](#_ENREF_5_5)] |
|  | Former cancer | | First record at least 2 months before HNSCC diagnosis |  |
|  | Second synchronous cancer | | First record within the last 2 months before HNSCC diagnosis and the first 6 months after HNSCC diagnosis |  |
|  | Second metachronous cancer | | First record after 6 months |  |
|  | Lung cancer | | C33-C34 |  |
|  | Esophageal cancer | | C15 |  |
|  | Other cancer | | Cxx other than HNSCC, lung cancer, esophageal cancer, or lymph node/distant metastasis |  |
| Any distant metastasis after diagnosis | | | C78-C79 |  |
| **Comorbidities other than cancer** | | | | |
| Charlson comorbidities other than cancer | | | ICD-10 coding algorithm validated for each comorbidity | [[8-11](#_ENREF_5_8)] |
|  | Including Human Immunodeficiency Virus (HIV) infection or Acquired Immune Deficiency Syndrome (AIDS) | | Z21; B20-B24 |  |
| Depression or suicide attempt | | | F32-F33; X6, X7, X80-X84 | [[12](#_ENREF_5_12),[13](#_ENREF_5_13)] |
| **End-of-life characteristics** | | | | |
| Palliative care | | | Z51.5 |  |

Reference List

1. Gatta G, Botta L, Sanchez MJ, Anderson LA, Pierannunzio D, et al. (2015) Prognoses and improvement for head and neck cancers diagnosed in Europe in early 2000s: The EUROCARE-5 population-based study. Eur J Cancer 51: 2130-2143.

2. Amin MB, Edge S, Greene F, Byrd DR, Brookland RK, et al. (2017) AJCC Cancer Staging Manual, 8th ed. New-York: Springer-Verlag.

3. Gregoire V, Lefebvre JL, Licitra L, Felip E, Group E-E-EGW (2010) Squamous cell carcinoma of the head and neck: EHNS-ESMO-ESTRO Clinical Practice Guidelines for diagnosis, treatment and follow-up. Ann Oncol 21 Suppl 5: v184-186.

4. Institut National du Cancer (INCa) (2013) Algorithme de sélection des hospitalisations liées à la prise en charge du cancer dans les bases nationales d'activité hospitalière de court séjour « algorithme cancer » [Algorithm to select cancer-related hospitalizations in the French National Hospital Discharge (PMSI) database]. Boulogne-Billancourt: INCa.

5. Working Group R (2005) International rules for multiple primary cancers (ICD-0 third edition). Eur J Cancer Prev 14: 307-308.

6. Howlader N, Ries LA, Mariotto AB, Reichman ME, Ruhl J, et al. (2010) Improved estimates of cancer-specific survival rates from population-based data. J Natl Cancer Inst 102: 1584-1598.

7. Jegu J, Colonna M, Daubisse-Marliac L, Tretarre B, Ganry O, et al. (2014) The effect of patient characteristics on second primary cancer risk in France. BMC Cancer 14: 94.

8. Charlson ME, Pompei P, Ales KL, MacKenzie CR (1987) A new method of classifying prognostic comorbidity in longitudinal studies: development and validation. J Chronic Dis 40: 373-383.

9. Quan H, Sundararajan V, Halfon P, Fong A, Burnand B, et al. (2005) Coding algorithms for defining comorbidities in ICD-9-CM and ICD-10 administrative data. Med Care 43: 1130-1139.

10. Quan H, Li B, Couris CM, Fushimi K, Graham P, et al. (2011) Updating and validating the Charlson comorbidity index and score for risk adjustment in hospital discharge abstracts using data from 6 countries. Am J Epidemiol 173: 676-682.

11. Bannay A, Chaignot C, Blotiere PO, Basson M, Weill A, et al. (2016) The Best Use of the Charlson Comorbidity Index With Electronic Health Care Database to Predict Mortality. Med Care 54: 188-194.

12. GBD 2015 Mortality and Causes of Death Collaborators (2016) Global, regional, and national life expectancy, all-cause mortality, and cause-specific mortality for 249 causes of death, 1980-2015: a systematic analysis for the Global Burden of Disease Study 2015. Lancet 388: 1459-1544.

13. Schwarzinger M, Pollock BG, Hasan OSM, Dufouil C, Rehm J, et al. (2018) Contribution of alcohol use disorders to the burden of dementia in France 2008-13: a nationwide retrospective cohort study. Lancet Public Health 3: e124-e132.

## S2 Table: EPICORL study population

| **All adults residing in metropolitan France and discharged in 2008-2012 (French National Hospital Discharge database)** | | 27 284 709 |
| --- | --- | --- |
| **Selection: head and neck cancer (ICD-10: C00-C14; C30-C32) in 2008-2012** | | 134,324 (0.49) |
| **Exclusion: any patient recorded with non-squamous cell carcinoma** | | 2,359 (1.76) |
|  | Salivary glands (ICD-10: C07-C08) | 1,427 (1.06) |
|  | Skin cancer of lip or face (ICD-10: C44.0-C44.3) | 708 (0.53) |
|  | Middle ear (ICD-10: C30.1) | 152 (0.11) |
|  | Kaposi sarcoma of the head and neck (ICD-10: C46.2; C46.70) | 52 (0.04) |
|  | Melanoma of lip or face (ICD-10: C43.0-C43.3) | 47 (0.03) |
| **Inclusion: HNSCC (ICD-10: C00-C06; C09-C14; C30.0; C31; C32) in 2008-2012** | | 131,965 (0.48) |
| **In-hospital deaths in 2008-2012** | | 46,463 (35.2) |

ICD-10: International Classification of Diseases and Related Health Problems, 10^th^ revision

## S3 Table: Identification of deaths attributable to HNSCC in the PMSI database, overall and by year of death (France, 2008-2012)

| **Deaths attributable to HNSCC** | | **Total 2008-2012** | **2008** | **2009** | **2010** | **2011** | **2012** |
| --- | --- | --- | --- | --- | --- | --- | --- |
| **Main analysis (in-hospital deaths)** | | 41,503 (100) | 7,251 (17.5) | 8,106 (19.5) | 8,598 (20.7) | 8,738 (21.1) | 8,810 (21.2) |
| Deaths attributable to HNSCC | |  |  |  |  |  |  |
|  | Probable | 28,254 (68.1) | 3,707 (51.1) | 5,475 (67.5) | 6,094 (70.9) | 6,424 (73.5) | 6,554 (74.4) |
|  | Possible | 13,249 (31.9) | 3,544 (48.9) | 2,631 (32.5) | 2,504 (29.1) | 2,314 (26.5) | 2,256 (25.6) |
| Stage at initial treatment | |  |  |  |  |  |  |
|  | Distant metastasis stage | 10,882 (26.2) | 2,763 (38.1) | 2,287 (28.2) | 2,079 (24.2) | 1,921 (22.0) | 1,832 (20.8) |
|  | Locally advanced stage | 27,467 (66.2) | 4,405 (60.8) | 5,333 (65.8) | 5,777 (67.2) | 5,960 (68.2) | 5,992 (68.0) |
|  | Early stage | 3,154 (7.6) | 83 (1.1) | 486 (6.0) | 742 (8.6) | 857 (9.8) | 986 (11.2) |
| Relapse in follow-up | | 16,233 (39.1) | 710 (9.8) | 2,956 (36.5) | 3,785 (44.0) | 4,279 (49.0) | 4,503 (51.1) |
| **Sensitivity analysis (overall deaths)^a^** | | 51,129 (100) | 8,887 (17.4) | 9,886 (19.3) | 10,309 (20.2) | 10,673 (20.9) | 11,374 (22.3) |
| Deaths attributable to HNSCC | |  |  |  |  |  |  |
|  | Probable | 34,196 (66.9) | 4,572 (51.5) | 6,600 (66.8) | 7,214 (70.0) | 7,692 (72.1) | 8,118 (71.4) |
|  | Possible | 16,933 (33.1) | 4,315 (48.5) | 3,286 (33.2) | 3,095 (30.0) | 2,981 (27.9) | 3,256 (28.6) |
| Stage at initial treatment | |  |  |  |  |  |  |
|  | Early stage | 3,814 (7.5) | 105 (1.2) | 574 (5.8) | 898 (8.7) | 1,014 (9.5) | 1,223 (10.8) |
|  | Locally advanced stage | 34,116 (66.7) | 5,388 (60.6) | 6,475 (65.5) | 6,934 (67.3) | 7,392 (69.2) | 7,927 (69.7) |
|  | Distant metastasis stage | 13,199 (25.8) | 3,394 (38.2) | 2,837 (28.7) | 2,477 (24.0) | 2,267 (21.2) | 2,224 (19.5) |
| Relapse in follow-up | | 19,358 (37.9) | 884 (10.0) | 3,452 (34.9) | 4,416 (42.8) | 5,080 (47.6) | 5,526 (48.6) |

**^a^** In-hospital deaths or deaths imputed outside hospital in patients lost to follow-up at hospital.

## S4 Table: Characteristics of deaths attributable to HNSCC only identified in the PMSI database (France, 2008–2012)

| **Characteristics of death**  **attributable to HNSCC** | | **Main analysis (in-hospital deaths)** | **Sensitivity analysis (overall deaths)^a^** |
| --- | --- | --- | --- |
|  |  | N=41,503 | N=51,129 |
| Multiple primary HNSCC | | 5,359 (12.9) | 6,378 (12.5) |
|  | Personal history of HNSCC | 1,945 (4.7) | 2,300 (4.5) |
|  | Second synchronous HNSCC | 3,575 (8.6) | 4,260 (8.3) |
|  | Second metachronous HNSCC | 1,202 (2.9) | 1,371 (2.7) |
| Primary cancer other than HNSCC | | 17,673 (42.6) | 20,969 (41.0) |
|  | Lung cancer | 6,699 (16.1) | 7,944 (15.5) |
|  | Esophageal cancer | 3,206 (7.7) | 3,746 (7.3) |
|  | Other primary cancer | 8,192 (19.7) | 9,785 (19.1) |
|  | Former primary cancer | 4,051 (9.8) | 4,754 (9.3) |
|  | Second synchronous cancer | 9,232 (22.2) | 11,052 (21.6) |
|  | Second metachronous cancer | 4,390 (10.6) | 5,163 (10.1) |
| Any distant metastasis after diagnosis | | 19,621 (47.3) | 23,451 (45.9) |
| Comorbidities | | 26,646 (64.2) | 32,277 (63.1) |
|  | HIV/AIDS | 190 (0.5) | 224 (0.4) |
|  | Myocardial infarction | 2,247 (5.4) | 2,714 (5.3) |
|  | Congestive heart failure | 5,978 (14.4) | 7,207 (14.1) |
|  | Peripheral vascular disease | 6,277 (15.1) | 7,548 (14.8) |
|  | Cerebrovascular disease | 4,500 (10.8) | 5,380 (10.5) |
|  | Dementia | 1,423 (3.4) | 1,931 (3.8) |
|  | Chronic pulmonary disease | 10,865 (26.2) | 13,130 (25.7) |
|  | Rheumatologic disease | 279 (0.7) | 341 (0.7) |
|  | Peptic ulcer disease | 1,427 (3.4) | 1,727 (3.4) |
|  | Hemiplegia or paraplegia | 2,289 (5.5) | 2,670 (5.2) |
|  | Renal disease | 2,949 (7.1) | 3,561 (7.0) |
|  | Mild liver disease | 2,388 (5.8) | 2,869 (5.6) |
|  | Moderate or severe liver disease | 1,872 (4.5) | 2,143 (4.2) |
|  | Diabetes | 5,738 (13.8) | 6,920 (13.5) |
|  | Depression | 4,432 (10.7) | 5,418 (10.6) |
|  | Suicide attempt | 346 (0.8) | 417 (0.8) |
| End-of-life characteristics | |  |  |
|  | Palliative care | 28,538 (68.8) | 31,744 (62.1) |
|  | Place of death: |  |  |
|  | Comprehensive cancer care center | 2 294 (5.5) | 2 294 (4.5) |
|  | Public teaching hospital | 7 169 (17.3) | 7 169 (14.0) |
|  | Private clinic | 8 772 (21.1) | 8 772 (17.2) |
|  | Public local hospital | 23 268 (56.1) | 23 268 (45.5) |
|  | Home (death imputed outside hospital) | -- | 9,626 (18.8) |

**^a^** In-hospital deaths or deaths imputed outside hospital in patients lost to follow-up after hospital discharge.

Results are presented as n (%). HNSCC: head and neck squamous cell carcinoma; HIV: Human Immunodeficiency Virus infection; AIDS: Acquired Immune Deficiency Syndrome.
