# Supplementary material for: Factors associated with under-reporting of head and neck squamous cell carcinoma in cause-of-death records: A comparative study of two national databases in France from 2008 to 2012
Source: PLoS One. 2021 Feb 3;16(2):e0246252. doi: 10.1371/journal.pone.0246252 (PMC7857613; doi:10.1371/journal.pone.0246252)
Supplement: Attachment — Submitted filename: Major Revisions_PONE-D-20-25023.docx [file pone.0246252.s002.docx]

**August 28, 2020**

**Manuscript Number: PONE-D-20-25023**

**“Underlying cause-of-death statistics underestimate the burden of head and neck squamous cell carcinoma: a nationwide hospital study in France, 2008 - 2012”**

Dear Dr Brecht Devleesschauwer,

I deeply appreciate your kind consideration of me to review this manuscript. I reviewed it with great interest.

Authors have tried to highlight the underreporting of causes of death of head and neck squamous cell carcinoma (HNSCC) by comparing two national databases and identified some potential factors associated with that. The approach used in this study is interesting. The evidence of this study is of great importance to improve the coding of related deaths that would help to calculate the burden of disease estimates correctly.

The authors did not actually estimate the burden of death statistics of HNSCC but highlighted the under-reporting of death statistics. Therefore, I propose to revise the title of the manuscript such as “*Factors associated with under-reporting of death statistics of HNSCC: A comparative study of two national databases in France from 2008 to 2012.”*

The authors have performed a multivariate Poisson regression analysis to identify the factors associated with under-reporting of HNSCC death statistics. Overall analysis and the results are robust. However, this manuscript needs to improve the quality of writing throughout the manuscript and the structure of methods, results and discussion sections. Most of the sentences are too long and should split in short to make it clear and more comprehensible. Therefore, the authors should ask a native English speaker for its proofreading. Therefore, I proposed “major revisions” to improve the quality of writing of this manuscript. Please find my comments below this letter.

Sincerely,

Romana Haneef

**Reviewer’s comments to authors**

**Formatting**

Page numbers and line numbers are missing. The spacing between the references numbers is not appropriate. Please update the manuscript according to the guidelines of the journal.

**Title:**

The authors did not estimate the burden of death statistics of HNSCC but highlight the under-reporting of death statistics. Therefore, I propose to revise the title of the manuscript such as “*Factors associated with under-reporting of death statistics of HNSCC: A comparative study of two national databases in France from 2008 to 2012.”*

**Abstract**

Please add the following information:

- *Objective:* The authors can update and add another objective as follows: *“Our objectives were to compare the reporting of death attributable to HNSCC from two national databases in France and to identify the underlying factors associated with under-reporting.”*
- *Methods:* Authors used the word “The determinants of *missing* HNSCC…, it is better to replace it with *under-reporting.*
- *Results:* Please revise the sentence structures for the results section.
- *Conclusions: P*lease revise the first sentence according to the updated title.

**Introduction**

- In the first paragraph, last sentence “to our knowledge […]”, authors used exposure to tobacco and alcohol account for larger population attributable fractions of HNSCC but did not take into account these factors in their analysis. It is unclear to me mention it here. I propose to remove it and rephrase it.
- Please replace the word “combining” with “linking” individual records […] at the beginning of the second paragraph.
- Please revise the text to improve the level of writing and avoid using words like instead, around, possibly.

**Materials and methods**

- Please add as a sub-heading the study design (part of the text from sub-section of the *definition of HNSCC attributable deaths at hospital*) and study population (inclusion and exclusion criteria from S2 table and cite S2 table under this section) just before the data sources.
- Please also add the definition of “unlikely cause of death” at the end of the sub-section of the *definition of HNSCC attributable deaths at hospital).*
- Please revise the text of the last paragraph of the sub-section *of “characteristics of HNSCC attributable deaths at hospital”* and mention the “end of life characteristics” as 1. palliative care and 2. place of death (under this characteristic, authors can report deaths recorded at different places). This description should correspond to the table, which is not numbered (I suppose, table 4).
- Please specify death at six age groups in the main text under the section of *UCoD statistics for HNSCC.*
- Please add the sub-section of *study outcomes* before the *statistical analysis* (the first paragraph of statistical analysis could be moved to the study outcome section).
- Please revise the text to improve the level of writing

**Results**

1. The authors can start the result section by reporting the *“General characteristics of deaths attributable to HNSCC according to two national databases in France”* as table 1 rather than table 2.
   a. Please add the word “National”UCoD statistics in table 1 (currently table 2)
2. The second sub-section of results could be *“Death attributable to HNSCC at hospital”* as table 2.
   a. Under this section, please also report the “unlikely death attributable to HNSCC” in the main text.
   b. Authors can report the “relapse in the follow-up overall from 2008 to 2010 from S3table to the main text and in table 2 as well.
   c. Citation of S2 table is not appropriate in this section.
   d. Please remove the word “definition” from the legend of table 2 (currently table 1) and start with “Deaths attributable […]”.
   e. In figure 1, please report the respective percentages of probable and possible causes of death attributable to HNSCC. Currently, it is unclear, for example in 2008, under in-hospital death, 70% corresponds to both causes of death (probable and possible)? Is this figure based on the data from S3 table?
3. The last paragraph of sub-section “Comparison of HNSCC […]”, please replace the word “multiple primary HNSCC” with “decedent”, which does not correspond to the table S4table.
   a. Please update the title as follows: “Comparison of HNSCC […] with UCoD statistics”.
4. The last sub-section of the results is unclear with long sentences. I propose to revise it and split into short sentences to make it more comprehensible.
   a. Under this sub-section, a table is reported and not numbered. I guess it should be table 4. Please add the table number and heading/legend of this table.

**Discussion**

- The authors should revise the overall text of the discussion section without repeating the same results, avoid long sentences, report strengths and limitations in a more clear text.
- This section could be started such as “*The result of this study showed the underreporting of death attributable to HNSCC in national UCoD as compared to national hospital discharge database.”*
- Avoid using words such as massive, unexpectedly, rather, indeed, altogether, etc.
- Authors mentioned again the same phrase as reported in the *introduction section* *“To our knowledge […]”,* it is nuclear. Please explain the reason to repeat this sentence.

**Conclusions**

Please update the conclusions section and do not report the results in this section.
