# Supplementary material for: Factors associated with under-reporting of head and neck squamous cell carcinoma in cause-of-death records: A comparative study of two national databases in France from 2008 to 2012
Source: PLoS One. 2021 Feb 3;16(2):e0246252. doi: 10.1371/journal.pone.0246252 (PMC7857613; doi:10.1371/journal.pone.0246252)
Supplement: Attachment — Submitted filename: PONE-D-20-25023_Responses to Reviewers.docx [file pone.0246252.s003.docx]

**Reviewer’s comments to authors**

1. Is the manuscript technically sound, and do the data support the conclusions?
Reviewer #1: Yes

Reviewer #2: Yes

2. Has the statistical analysis been performed appropriately and rigorously?

Reviewer #1: Yes

Reviewer #2: Yes

3. Have the authors made all data underlying the findings in their manuscript fully available?
Reviewer #1: Yes

Reviewer #2: Yes

4. Is the manuscript presented in an intelligible fashion and written in standard English?
PLOS ONE does not copyedit accepted manuscripts, so the language in submitted articles must be clear, correct, and unambiguous. Any typographical or grammatical errors should be corrected at revision, so please note any specific errors here.

Reviewer #1: No

Reviewer #2: Yes

**The revised manuscript has been proofread by a native English speaker.**

## Reviewer #1 Romana Haneef

Dear Dr Brecht Devleesschauwer,

I deeply appreciate your kind consideration of me to review this manuscript. I reviewed it with great interest.

Authors have tried to highlight the underreporting of causes of death of head and neck squamous cell carcinoma (HNSCC) by comparing two national databases and identified some potential factors associated with that. The approach used in this study is interesting. The evidence of this study is of great importance to improve the coding of related deaths that would help to calculate the burden of disease estimates correctly.

The authors did not actually estimate the burden of death statistics of HNSCC but highlighted the under-reporting of death statistics. Therefore, I propose to revise the title of the manuscript such as “Factors associated with under-reporting of death statistics of HNSCC: A comparative study of two national databases in France from 2008 to 2012.”

The authors have performed a multivariate Poisson regression analysis to identify the factors associated with under-reporting of HNSCC death statistics. Overall analysis and the results are robust. However, this manuscript needs to improve the quality of writing throughout the manuscript and the structure of methods, results and discussion sections. Most of the sentences are too long and should split in short to make it clear and more comprehensible. Therefore, the authors should ask a native English speaker for its proofreading. Therefore, I proposed “major revisions” to improve the quality of writing of this manuscript. Please find my comments below this letter.

**Thank you for your positive appraisal of our study and the time taken to help improving the clarity of the manuscript. We have modified the title as suggested and the revised manuscript has been proofread.**

Formatting

Page numbers and line numbers are missing. The spacing between the references numbers is not appropriate. Please update the manuscript according to the guidelines of the journal.

**We are sorry for the inconvenience for the reviewing process. In the revised manuscript, we have added page numbers and line numbers per page. It seems double-spacing was already used throughout the manuscript, including for the references.**

Title

The authors did not estimate the burden of death statistics of HNSCC but highlight the under-reporting of death statistics. Therefore, I propose to revise the title of the manuscript such as “*Factors associated with under-reporting of death statistics of HNSCC: A comparative study of two national databases in France from 2008 to 2012.”*

**We agree with the reviewer and the title has been changed accordingly into “Factors associated with under-reporting of head and neck squamous cell carcinoma in cause-of-death records: a comparative study of two national databases in France from 2008 to 2012”.**

Abstract

Please add the following information:

- *Objective:* The authors can update and add another objective as follows: *“Our objectives were to compare the reporting of death attributable to HNSCC from two national databases in France and to identify the underlying factors associated with under-reporting.”*

**We agree with the reviewer and objectives have been modified accordingly in the abstract.**

- *Methods:* Authors used the word “The determinants of *missing* HNSCC…, it is better to replace it with *under-reporting.*

**We agree with the reviewer that it is better to use the same terminology and “determinants of missing HNSCC” has been replaced by “factors associated with under-reporting of HNSCC” throughout the revised manuscript.**

- *Results:* Please revise the sentence structures for the results section.

**The revised manuscript has been proofread.**

- *Conclusions: P*lease revise the first sentence according to the updated title.

**This has been done in the revised manuscript.**

Introduction

- In the first paragraph, last sentence “to our knowledge […]”, authors used exposure to tobacco and alcohol account for larger population attributable fractions of HNSCC but did not take into account these factors in their analysis. It is unclear to me mention it here. I propose to remove it and rephrase it.

**We agree with the reviewer and we removed this from the Introduction in the revised manuscript. The ideas are now taken up in the Discussion.**

- Please replace the word “combining” with “linking” individual records […] at the beginning of the second paragraph.

**This has been corrected in the revised manuscript.**

- Please revise the text to improve the level of writing and avoid using words like instead, around, possibly.

**We have rewritten the second paragraph to improve clarity in the revised manuscript that has been proofread.**

Materials and methods

- Please add as a sub-heading the study design (part of the text from sub-section of the *definition of HNSCC attributable deaths at hospital*) and study population (inclusion and exclusion criteria from S2 table and cite S2 table under this section) just before the data sources.

**Following the reviewer’s suggestion, we have added a *Study design* section at the beginning of Material and Methods in the revised manuscript. In addition, inclusion and exclusion criteria (S2 Table), which are specific to the retrospective analysis of the national hospital discharge database have been transferred from the Results to the corresponding section in the Material and Methods (*Identification of HNSCC-attributable deaths at hospital*).**

- Please also add the definition of “unlikely cause of death” at the end of the sub-section of the *definition of HNSCC attributable deaths at hospital).*

**This has been added in the revised manuscript.**

- Please revise the text of the last paragraph of the sub-section *of “characteristics of HNSCC attributable deaths at hospital”* and mention the “end of life characteristics” as 1. palliative care and 2. place of death (under this characteristic, authors can report deaths recorded at different places). This description should correspond to the table, which is not numbered (I suppose, table 4).

**This has been modified in the revised manuscript and “place of death” is now described in the Material and Methods, consistent with the terminology used in Table 3. All Tables were numbered in the submitted manuscript and there was no Table 4.**

- Please specify death at six age groups in the main text under the section of *UCoD statistics for HNSCC.*

**We have specified age groups in the revised manuscript.**

- Please add the sub-section of *study outcomes* before the *statistical analysis* (the first paragraph of statistical analysis could be moved to the study outcome section)

**This has been done in the revised manuscript.**

- Please revise the text to improve the level of writing

**The revised manuscript has been proofread.**

Results

1. The authors can start the result section by reporting the *“General characteristics of deaths attributable to HNSCC according to two national databases in France”* as table 1 rather than table 2.

**We would prefer to keep the initial order of presentation of the results (main text and Tables 1 and 2) in the revised manuscript. It seems more appropriate first to describe the HNSCC-attributable in-hospital deaths (Table 1) and then to compare these in-hospital deaths with those extracted from the national UCoD statistics (Table 2).**

a. Please add the word “National” UCoD statistics in table 1 (currently table 2)

**This has been added in the revised manuscript.**

2. The second sub-section of results could be *“Death attributable to HNSCC at hospital”* as table 2.

**Please see above about reordering of the presentation of results.**

a. Under this section, please also report the “unlikely death attributable to HNSCC” in the main text.

**This has been added in the revised manuscript.**

b. Authors can report the “relapse in the follow-up overall from 2008 to 2010 from S3table to the main text and in table 2 as well.

**This has been added in the revised manuscript.**

c. Citation of S2 table is not appropriate in this section.

**This has been removed in the revised manuscript.**

d. Please remove the word “definition” from the legend of table 2 (currently table 1) and start with “Deaths attributable […]”.

**This has been done in the revised manuscript**

e. In figure 1, please report the respective percentages of probable and possible causes of death attributable to HNSCC. Currently, it is unclear, for example in 2008, under in-hospital death, 70% corresponds to both causes of death (probable and possible)? Is this figure based on the data from S3 table?

**We agree with the reviewer that Figure 1, which presents annual detection rates of HNSCC-attributable in-hospital deaths identified in the national UCoD statistics could also be used to document the proportion of probable and possible in-hospital HNSCC deaths (presented in S3 Table). In line with the reviewer’s suggestion, we have clarified this in the revised manuscript. Figure 1 was removed from the first sub-section of the results (about probable/possible cause of death) to the second sub-section (about detection rates of HNSCC-attributable in-hospital deaths identified in the national UCoD statistics). Citation of Figure 1 in the first sub-section of results (about probable/possible cause of death) has been replaced by citation of S3 Table.**

3. The last paragraph of sub-section “Comparison of HNSCC […]”, please replace the word “multiple primary HNSCC” with “decedent”, which does not correspond to the table S4table.

**This has been corrected in the revised manuscript.**

a. Please update the title as follows: “Comparison of HNSCC […] with UCoD statistics”.

**This has been corrected in the revised manuscript.**

4. The last sub-section of the results is unclear with long sentences. I propose to revise it and split into short sentences to make it more comprehensible.

**This sub-section has been shortened to improve clarity in the revised manuscript.**

a. Under this sub-section, a table is reported and not numbered. I guess it should be table 4. Please add the table number and heading/legend of this table.

**This was actually the second part (elasticities) of Table 3. The two parts (relative risks and elasticities) of Table 3 have been reconciled when reformatting the revised manuscript.**

Discussion

- The authors should revise the overall text of the discussion section without repeating the same results, avoid long sentences, report strengths and limitations in a more clear text.

**We somewhat disagree on the first point of the reviewer as it seems better to elaborate the discussion starting from the main results. In addition, while we agree that Introduction/M&M/Results should be as concise as possible (see agreements with the previous comments of the reviewer), we do much appreciate having no words count limit in *Plos One* to put results into perspective. The revised manuscript has been proofread.**

- This section could be started such as “*The result of this study showed the underreporting of death attributable to HNSCC in national UCoD as compared to national hospital discharge database.”*

**This has been done in the revised manuscript and the first paragraph of the Discussion has been rewritten.**

- Avoid using words such as massive, unexpectedly, rather, indeed, altogether, etc.

**The revised manuscript has been proofread.**

- Authors mentioned again the same phrase as reported in the *introduction section* *“To our knowledge […]”,* it is nuclear. Please explain the reason to repeat this sentence.

**This has been removed in the revised manuscript.**

Conclusions

Please update the conclusions section and do not report the results in this section.

**This has been done in the revised manuscript.**

## Reviewer #2: Ms Michelle Gourley

Overall the paper is well written and the analyses presented is clear and strongly support the conclusions reached.

**Thank you very much for your positive appraisal of our study.**

Some minor comments to improve the paper are outlined below:

- The paper doesn't make it clear whether deaths with an associated cause of death recorded as HNSCC have been looked at. Analyses of Australian deaths data suggests that HNSCC deaths would increase by around one-third (33%) if associated causes of death are included. Can this be looked at in the French mortality data? If not, or if no deaths are recorded with HNSCC as an associated cause, then suggest making this more explicit in the paper.

**National UCoD statistics only provide summary data for any selected UCoD (by year, gender, age group, region of residence). For this reason, we had no access to individual MCoD (multiple causes of death) nor to the number of HNSCC that may be coded as an associated cause of death. Following the reviewer’s comment, we have explicitly clarified that we had no access to MCoD data in the revised manuscript and discussed this point among the limitations of our study.**

- The statistics reported in the paper do not include a variance measure (ie confidence intervals). Is it possible to include these, or if not, provide a reason why these have not been included or are deemed not to be needed for the analyses presented.

**We did not calculate confidence intervals for summary statistics presented in Table 1 and 2 (counts), Figure 1 (counts), and Figures 2 to 5 (ASMR) because the summary statistics are related to observations at the national level and it was not relevant to account for sampling error. Otherwise, 95% confidence intervals were presented for all coefficients estimated in the multivariate Poisson regression (Table 3).**

- Paper could benefit from a gramma check to fix minor grammatical errors eg in the abstract results 'associated to' should be 'associated with'

**This has been corrected in the revised manuscript that has been also proofread.**
