# Supplementary material for: Factors associated with under-reporting of head and neck squamous cell carcinoma in cause-of-death records: A comparative study of two national databases in France from 2008 to 2012
Source: PLoS One. 2021 Feb 3;16(2):e0246252. doi: 10.1371/journal.pone.0246252 (PMC7857613; doi:10.1371/journal.pone.0246252)
Supplement: Attachment — Submitted filename: PONE-D-20-25023_R2 Responses to Reviewers.docx [file pone.0246252.s004.docx]

**Comments to the Author**

1. If the authors have adequately addressed your comments raised in a previous round of review and you feel that this manuscript is now acceptable for publication, you may indicate that here to bypass the “Comments to the Author” section, enter your conflict of interest statement in the “Confidential to Editor” section, and submit your "Accept" recommendation.

Reviewer #1: All comments have been addressed

Reviewer #2: All comments have been addressed

2. Is the manuscript technically sound, and do the data support the conclusions?

Reviewer #1: Yes

Reviewer #2: Yes

3. Has the statistical analysis been performed appropriately and rigorously?

Reviewer #1: Yes

Reviewer #2: Yes

4. Have the authors made all data underlying the findings in their manuscript fully available?

Reviewer #1: Yes

Reviewer #2: Yes

5. Is the manuscript presented in an intelligible fashion and written in standard English?

PLOS ONE does not copyedit accepted manuscripts, so the language in submitted articles must be clear, correct, and unambiguous. Any typographical or grammatical errors should be corrected at revision, so please note any specific errors here.

Reviewer #1: (No Response)

Reviewer #2: Yes

**Reviewer #2 who is a native English speaker found that both manuscript and R1 were “presented in an intelligible fashion and written in standard English”. Following the first comments of reviewer #1, the revised version of the manuscript (R1) has been entirely proofread by a native English speaker. We thank reviewer #1 for her continued scrutiny and all her comments that were taken into account, although we do not believe that re-asking that R2 is proofread by a native English speaker is necessary at this stage.**

**Reviewer #1: Reviewer’s comments to authors**

Abstract

• Results: Please add the word “national” before UCoD records [..] in the first phrase.

**This has been added in the R2 version of the manuscript.**

Materials and methods

• May be revise the title of the sub-section as “characteristics of HNSCC attributable deaths in national hospital discharge database (PMSI)”.

**This has been modified in the R2 version of the manuscript into “characteristics of HNSCC attributable deaths in the national hospital discharge database” as subtitles should be better kept short and reference to “PMSI” is provided at all occurrences in the text (see below).**

• Please add one sentence to introduce PMSI database in this section and explicit the abbreviation (programme de médicalisation des systèmes d’information).

**This was already done in the R1 version of the manuscript (lines 3-4 page 4).**

• It is better to use “national hospital discharge database (PMSI)” than only PMSI. In France, it is well known but in general using this abbreviation may be confusing. Therefore, I suggest adding, “national hospital discharge database and mention in bracket the PMSI”. Please update it throughout the manuscript.

**We agree with the reviewer. This has been modified in the R2 version of the manuscript except in subtitles and Table/Figure titles where the mention of “(PMSI)” has been removed (see above).**

Results

• Please revise the title of the section as suggested above in the method section as “Deaths attributable to HNSCC in national hospital discharge database (PMSI)”.

**This has been modified in the R2 version of the manuscript (see above).**

• Update the title of table 1 as well.

**This has been modified in the R2 version of the manuscript (see above).**

Discussion

This section needs some revisions and please check with an English native speaker for the proofreading of this section. Some suggestion to update the followings items:

**Please see above our overall comment.**

• Please update the sentence as “This study showed significant under-reporting […]”

**This has been modified in the R2 version of the manuscript.**

• First paragraph, last sentence: « […] national UCoD records was less than half of the PMSI database. »

**This has been modified in the R2 version of the manuscript.**

• Second paragraph, last sentence, could split in two sentences as follows: « This situation seems particularly critical for HNSCC-attributable deaths, since exposure to tobacco and alcohol are major risk factors for HNSCC. Moreover, multiple other causes of death that are declared in death certificates, may be responsible for that. »

**We agree with the reviewer. This has been clarified in the R2 version of the manuscript.**

• Third paragraph, in second sentence, please remove the word “consistently”.

**This has been removed in the R2 version of the manuscript.**

• On page 27

o line 2-3, please remove the word “taken together” and start the sentence with “These findings […].

**This has been removed in the R2 version of the manuscript.**

o Line 6, please remove “U.S.”.

**This has been removed in the R2 version of the manuscript.**

o Line 8-9, update the sentence as follows: “HNSCC-attributable deaths in PMSI database were considered as the rule than exception […].

**We disagree with the reviewer as this point was already discussed in the previous paragraph.**

o Line 13, please remove the word “generally”.

**This has been removed in the R2 version of the manuscript.**

o Iine 16-17, please remove the work “again” and “in general”.

**This has been removed in the R2 version of the manuscript.**

Conclusions

• Please remove the word “in particular”.

**We disagree with the reviewer as our results may apply to cancer deaths in general and HNSCC in particular.**

**Reviewer #2: The authors have adequately addressed my previous comments.**

One additional comment to consider is to add to the discussion that some of the head and neck SCC deaths may be hidden/included in the group of ICD 10 codes C76-C80 Malignant neoplasms of ill-defined, secondary and unspecified site. This could be the case where the certifying doctor has not provided specific details about the site and type of cancer on the medical certificate of cause of death.

**We agree with the reviewer. This was already discussed in the third paragraph: “We also found that any distant metastasis recorded after HNSCC diagnosis (47%) was associated with an increased risk of HNSCC being under-reported in UCoD records [13]” and fifth paragraph: “Since lung cancer was the most frequent second primary cancer (16.1%), and may arise from metastatic spread of HNSCC [2], our findings suggest that certifying physicians usually simplify the “chain of events leading directly to death” by reporting lung cancer as the UCoD rather than the primary HNSCC site”.**
